# Dr. Henry Granger Piffard

**Published:** 1910-07

**Authors:** 


### OBITUARY

---

Dr. Henry Granger Piffard, of New York, professor emeritus of dermatology in New York University and president of the alumni association of that institution, died June 8, 1910, at his home, No. 256 West 57th street, of pneumonia. For forty years Dr. Piffard had practised his profession in New York. He was considered an expert on skin diseases, and had written a number of books or monographs on that subject. At the time of his death he was consulting surgeon at the City Hospital.

He was born at Piffard, N. Y., in 1842, the son of David and Ann Matilda Haight Piffard. He attended New York University, from which he was graduated with the class of '62, and obtained his medical degree from the College of Physicians and Surgeons. In 1899 his alma mater conferred on him the degree of LL. D.

In 1868 Dr. Piffard married Helen Hart Strong, who, with one son and a daughter, survives him. He had been a professor in New York University since 1875.

